# A user-informed perspective of the toxicological data gap in India’s cannabis landscape

**DOI:** 10.3389/ftox.2025.1734313

**Published:** 2026-01-15

**Authors:** Muzafar Riyaz

**Affiliations:** Division of Entomology, Sher-e-Kashmir University of Agricultural Sciences and Technology, Jammu, India

**Keywords:** adulteration, cannabis regulation, harm reduction, illicit market, India, public health policy, toxicology, user-informed research

## Abstract

Clinical research on cannabinoids relies on purified compounds and controlled dosing, creating a data gap that ignores the realities of illicit markets. This perspective, informed by thirteen years of firsthand experience within India’s prohibited cannabis ecosystem, argues that unregulated products like Ganja and Charas pose significant, overlooked toxicological risks. These risks arise not primarily from cannabinoids themselves, but from unpredictable potency, pesticide contamination, and adulteration in the absence of quality control. Personal consumption patterns reveal that inconsistent products make precise dosing impossible and that standard clinical assessments fail to capture users’ sought-after effects, such as cognitive enhancement. To address this public health challenge, this paper calls for: 1) the chemical analysis of illicit products, 2) qualitative research on real-world use, 3) the development of user-centered outcome measures, and 4) ultimately, a transition from prohibition to regulation as the most effective intervention for consumer safety and informed choice.

## 1 Introduction

The past decade has witnessed an unprecedented surge in clinical research into purified cannabinoids like CBD for therapeutic use (Liu et al., 2021; Johansson et al., 2025). This research operates within a paradigm of strict control, using standardized compounds and precise doses (Cross and Cock, 2020; Hossain and Chae, 2024). However, this controlled model is a world apart from the unregulated reality of global cannabis consumption (Goodman et al., 2020; Wanke et al., 2022). Nowhere is this disconnect more hazardous than in prohibited markets like India, where consumers rely on traditional, unrefined products like Ganja and Charas, and face routine product variability, adulteration, and “dosing chaos” (Shrinet, 2022; Chithra et al., 2023).

This perspective is informed by over 13 years of firsthand experience within India’s illicit cannabis ecosystem; a unique vantage point that bridges scientific rigor with user-level reality. It argues that the illicit market is not an outlier but a critical case study exposing a severe toxicological data gap. The primary risks for millions of users stem not from cannabinoids themselves, but from the uncontrolled context of production and consumption, which renders safety data from clinical trials largely irrelevant. To bridge this gap, this article calls for: (1) regulating cannabis to ensure quality and reduce stigma; (2) studying user-reported psychoactive effects like cognitive enhancement; (3) systematically analyzing the illicit supply; and (4) providing harm reduction guidance where prohibition persists.

## 2 The illicit market as a source of uncontrolled toxicological risk and product variability

The illicit cannabis market in India should be understood not only as a legal failure but as a critical source of uncontrolled public health risk. In this environment, all standard consumer safeguards, such as quality control, standardized production, and accurate labelling are absent (Mackey and Liang, 2011). This absence creates a state of persistent product uncertainty for consumers. While clinical research deliberately controls for these variables to ensure internal validity, this very practice excludes the chaotic realities that determine the actual risks and effects for millions of users. The Indian context, where a deep-rooted cultural history of cannabis use coexists with stringent prohibition, provides a clear illustration of this disconnect. The personal observations detailed in the following sections are therefore more than anecdotes; they are direct evidence of the toxicological consequences of an unregulated market, underscoring why scientific inquiry must account for real-world use.

Clinical research relies on consistent, chemically characterized products. In stark contrast, the illicit market offers no such assurances (Bancroft and Reid, 2016; van der Gouwe et al., 2017). Consumers have no reliable information on potency (THC/CBD ratios) or purity, often relying on flawed proxies like texture or smell. My long-term use of Ganja provided a direct lesson in this uncertainty. Product quality was perpetually inconsistent. Batches suspected of being contaminated with pesticides or rodenticides consistently induced unpleasant headaches; an adverse effect likely unrelated to the cannabinoids themselves. Conversely, “clean” batches delivered the desired effects without such side effects. This stark contrast within the same product type underscores how unregulated production directly impacts user health. Concentrated products like Charas (hash/resin) presented an even greater challenge. The inability to predict the psychoactive strength of a given piece meant each use risked an overwhelmingly potent “rollercoaster” effect, far exceeding the desired or comfortable level. This demonstrates that risk is amplified in unrefined, concentrated derivatives.

This variability introduces toxicological risks absent from clinical research. The illicit supply chain can expose users to pesticides, heavy metals, microbial contaminants, and intentional adulterants with foreign substances to increase weight or mimic effects (Castiglioni and Zuccato, 2010; Giorgetti et al., 2020; Potter and Klein, 2020). While systematic studies in India are lacking, the structural incentives for such contamination are universal (Ghuman et al., 2023). Consequently, for consumers in illicit markets, the primary risk often shifts from the cannabinoids themselves to the uncontrolled context of production. Safety data from trials on pure compounds thus become largely irrelevant for this population.

## 3 The impossibility of dosing and its consequences

In clinical trials, doses are precise and measured (McClements, 2020; Perucca and Bialer, 2020). In India’s illicit market, this precision is non-existent. Dosing is based on crude estimations: a “finger-sized piece” of Charas or a certain number of Ganja cigarettes. These visual measures are meaningless when the potency of different batches varies wildly, as described in the previous section. The common practice of smoking adds further unpredictability, making the process the opposite of the controlled administration used in research.

The common clinical advice to “start low and go slow” is impossible to follow when the user does not know what “low” means. One must “start unknown and go blind.” My experience confirms this directly. With a batch of good-quality Ganja, a certain volume might produce a manageable, focused state. However, with a bad or adulterated batch, the same visual amount could cause pronounced sleepiness or headaches, completely disrupting the intended activity (Stang, 2023). This is not user error but a direct result of an inconsistent product. The danger is clear: an unexpectedly potent batch can lead to overconsumption, causing acute anxiety, paranoia, or impaired coordination (Hoch et al., 2015; Blanco et al., 2016). Without a predictable dose-response relationship, defining a safe consumption limit is impossible.

This “dosing chaos” also nullifies any reliable therapeutic potential. A user seeking relief from pain or insomnia cannot depend on a consistent effect. The same visual dose from one batch may help, while from another it may be ineffective or cause distressing psychoactive effects. This inconsistency fosters disillusionment with cannabis as a medicine and creates a stark divide between positive clinical trial results and the frustrating reality of illicit market use.

## 4 The illicit user’s narrative: a critical blind spot in research

Clinical research assesses cannabis with standardized scales for anxiety or pain (Williams et al., 2010). However, these tools often miss the nuanced effects that define real-world use. My primary motivation for using high-quality cannabis was not recreation but cognitive enhancement, a state of deep, fluid concentration that facilitated more efficient work. This desired effect falls outside the scope of standard clinical assessments. Similarly, negative outcomes from poor-quality batches were not generic ‘dysphoria’ but specific physical symptoms like headaches and lethargy.

This blind spot is widened by the clinical separation of drug effects from their real-world “set and setting.” In India’s prohibited context, use is shaped by social stigma (being derogatorily labelled “*charsi*” and “*ganjedi*”) and the stress of secrecy. This environment can compound anxiety, a modulation entirely absent from controlled trials. Consequently, clinical models risk a dual failure: they may underestimate the drug’s reinforcing properties, which can be tied to subtle cognitive benefits, and they may overlook its full therapeutic potential by not capturing user-reported outcomes like mental calm or holistic wellbeing. Therefore, advancing the science requires methods that systematically capture this lived experience. The interconnected issues of product variability, dosing impossibility, and unmeasured subjective effects together expose the limitations of a research model that ignores the illicit market. Bridging this gap is an urgent imperative for a relevant and comprehensive public health understanding of cannabis.

## 5 Conclusions and recommendations

Clinical research on purified cannabinoids creates a dangerous blind spot by ignoring the reality of illicit markets. In India, as demonstrated through 13 years of lived experience, the primary toxicological risks for consumers arise not from the plant’s natural compounds, but from the unregulated production environment: wildly unpredictable potency, contamination from pesticides or heavy metals, and deliberate adulteration with unknown substances. This lack of basic safeguards transforms consumption into a hazardous gamble, where any potential benefit is inextricably linked to significant and unpredictable health risks, a fundamental public health failure.

Closing this critical data gap and mitigating harm requires immediate, coordinated action on four fronts. First, public health policy must recognize that the most effective toxicological intervention is a transition from prohibition to a regulated legal market, which enables mandatory quality control, accurate labelling, and consumer protection. Second, the scientific paradigm must expand to include qualitative, user-centered research that captures the full spectrum of effects, such as cognitive enhancement, which drive real-world use but are invisible to standard clinical metrics. Third, chemical surveillance studies are urgently needed to systematically document the actual composition of the illicit supply, providing an evidence base for accurate risk communication. Finally, where prohibition persists, there is an ethical obligation to disseminate pragmatic harm reduction information to educate consumers on dosing unpredictability and safer practices. Together, these steps form the only viable path toward a science and policy framework that is both rigorous and relevant to the lived experiences of millions.

## Data Availability

The original contributions presented in the study are included in the article/supplementary material, further inquiries can be directed to the corresponding author.
